# Peristaltic Motion of Johnson-Segalman Fluid in a Curved Channel with Slip Conditions

**DOI:** 10.1371/journal.pone.0114168

**Published:** 2014-12-04

**Authors:** Sadia Hina, Meraj Mustafa, Tasawar Hayat

**Affiliations:** 1 Department of Mathematical Sciences, Fatima Jinnah Women University, Rawalpindi, Pakistan; 2 School of Natural Sciences (SNS), National University of Sciences and Technology (NUST), Islamabad, Pakistan; 3 Department of Mathematics, Quaid-I-Azam University 45320, Islamabad, Pakistan; 4 Department of Mathematics, Faculty of Science, King Abdulaziz University, Jeddah, Saudi Arabia; Northwestern Polytechnical University, China

## Abstract

Slip effects on the peristaltic transport of Johnson-Segalman fluid through a curved channel have been addressed. The influence of wall properties is also analyzed. Long wavelength and low Reynolds number assumptions have been utilized in the mathematical formulation of the problem. The equations so formed have been solved numerically by shooting method through computational software Mathematica 8. In addition the analytic solution for small Weissenberg number (elastic parameter) is computed through a regular perturbation method. An excellent agreement is noticed between the two solutions. The results indicate an increase in the magnitude of velocity with an intensification in the slip effect. Moreover the size and circulation of the trapped boluses increase with an increase in the slip parameter. Unlike the planar channel, the profiles of axial velocity are not symmetric about the central line of the channel.

## Introduction

Peristalsis is the form of fluid transport due to the waves travelling along the length of distensible tube/channel. This mechanism frequently occur in ureter for the transport of urine, in swallowing food through esophagus, in lymphatic vessels for the transport of lymph, in the vasomotion of small blood vessels, in roller and finger pumps and in heart-lung machine. Latham [1] and Jaffrin and Shapiro [2] have initially discussed the process of peristalsis in tube/channel. The influences of magnetic field and slip boundary condition on the peristaltic transport of viscous fluid have been studied by Ebaid [3]. Mekheimer and Elmaboud [4] examined the peristaltic motion of couple stress fluid in an annulus. The behavior of induced magnetic field on peristaltic transport of couple stress fluid in a planar channel has been addressed by Mekheimer [5]. Hydromagnetic peristaltic flow of Jeffrey fluid in an asymmetric channel has been investigated by Kothandapani and Srinivas [6]. Peristaltic flow of Newtonian fluid in an inclined asymmetric channel through porous medium has been reported by Kothandapani and Srinivas [7]. Hayat et al. [8] analyzed the effects of slip and temperature dependent viscosity on magnetohydrodynamic peristaltic flow of viscous fluid. Gad [9] investigated the effect of Hall current on the peristaltic flow of particle-fluid suspension. Influence of heat transfer and slip on peristaltic transport is analyzed by Hayat et al. [10]. Srinivas et al. [11] studied the mixed convective heat and mass transfer in an asymmetric channel with peristalsis. Effects of induced magnetic field on the peristaltic motion of nanofluid have been addressed by Mustafa et al. [12]. In another paper, Mustafa et al. [13] employed Keller-box method in computing numerical solutions for peristaltic transport of fourth grade fluid with Dufour and Soret effects.

The well known Navier-Stokes equations are incapable of describing the flow behavior of many complex fluids including clay coatings and suspensions, drilling muds, liquid detergents, oils and grease etc. To overcome this difficulty the researchers have proposed a variety of non-Newtonian fluid models. These mathematical models give rise to the differential equations which are highly nonlinear and more complicated than the Navier-Stokes equations. Different from the other fluid models, the Johnson-Segalman fluid model allows for non-monotonic variation in the shear stress with the increase/decrease in the rate of deformation for simple shear flow. Certain non-Newtonian fluids contain the interesting phenomenon of “ spurt”. The term “ Spurt” is used to describe abnormal increase in the volume throughput for a little increase in the driving pressure gradient. A different explanation of the “ Spurt” phenomenon based on the non-monotic relationship between stress and shear rate has also been described by some authors [14]. Hayat et al. [15] analyzed the peristaltic flow of Johnson-Segalman fluid in a planar channel. Elshahed and Haroun [16] studied the peristaltic motion of Johnson-Segalman fluid under the effect of magnetic field. The peristaltic transport of Johnson-Segalman fluid in an asymmetric channel has been discussed by Hayat et al. [17]. Wang et al. [18] studied the peristaltic motion of Johnson-Segalman fluid through a deformable tube. Nadeem and Akbar [19] studied the effects of induced magnetic field and heat and mass transfer on peristaltic flow of Johnson-Segalman fluid in a vertical asymmetric channel.

Peristaltic flow in channel/tube mainly occurs because of the flexibility of the walls. In existing literature, the reasonable attention is given to the peristaltic flows in a channel having compliant walls. Mittra and Prasad [20] analyzed the effects of wall properties on peristalsis. Davies and Carpenter [21] analyzed the stability of plane channel flow with compliant walls. Srivastava and Srivastava [22] studied the wall elasticity on peristaltic flow of particle-fluid mixture. Haroun [23] studied the compliant wall effects on peristalsis in an asymmetric channel. Radhakrishnamacharya and Srinivasulu [24] studied the effect of flexible walls on peristaltic transport with heat transfer. Muthu et al. [25] discussed the wall properties on the peristaltic flow of micropolar fluid in a circular cylindrical tube. Elnaby and Haroun [26] studied the effect of wall properties on peristaltic transport of a viscous fluid. Hayat et al. [27] and Hayat et al. [28] have seen the influence of compliant walls on the peristaltic flow of Johnson-Segalman and Jeffery fluids respectively. Peristaltic transport of Maxwell fluid in a channel with compliant walls is considered by Ali et al. [29]. Kothandapani and Srinivas [30] discussed the effects of wall properties on MHD peristaltic flow of viscous fluid in a channel with porous medium. This work is extended by Srinivas et al. [31] in the presence of slip effects. Srinivas and Kothandapani [32] have also examined the compliant wall effects on MHD peristaltic flow through a porous space. Hayat and Hina [33] explored the compliant wall effect on peristaltic flow of Maxwell fluid.

From the above discussion it is noticed that not many studies have been carried out on peristaltic mechanism in a curved channel. Sato et al. [34] discussed the peristaltic flow due to the transverse deflections of the walls of the curved channel. Ali et al. [35] studied the peristaltic flow analysis in a curved channel with long wavelength approximation. Ali et al. [36], [37] have extended the analysis of ref. [33] for a third grade fluid and heat transfer characteristics. Hayat et al. [38] discussed the peristaltic transport of Newtonian fluid in a curved channel with compliant walls. Hayat et al. [39] and Hina et al. [40] extended the the analysis of [38] for third grade and Johnson-Segalman fluids respectively. Combined heat and mass transfer effects on the peristaltic flow of Johnson-Segalman fluid have been explored by Hina et al. [41]. Influence of wall properties on the peristaltic flow of pseudoplastic fluid in a curved channel have been described by Hina et al. [42]. Peristaltic motion of third grade fluid with slip effects has been discussed by Hina et al. [43]. Hina et al. [44] also numerically investigated the peristaltic flow of nanofluid in a curved channel.

Peristaltic transport of Johnson-Segalman fluid with slip conditions has never been reported in the literature. The present work extends the flow analysis of ref. [35] from viscous to the Johnson-Segalman fluid with slip effects and wall properties. The fluid motion is considered in a curved channel due to the fact that most of the arteries and glandular ducts are curved. Low Reynolds number and long wavelength approximation have been used for the development of mathematical model. The numerical solutions have been computed via shooting method through computational software Mathematica. The analytic solutions are also obtained by a regular perturbation method. Graphs for the embedded flow quantities are portrayed and discussed to get the physical insight of the problem.

## Mathematical Formulation

**Figure 1:**
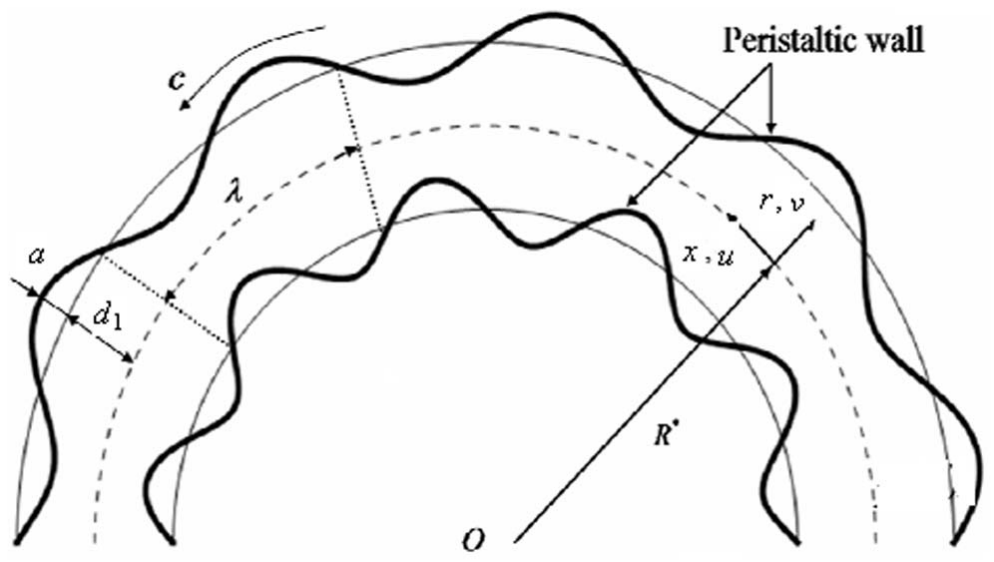
Physical sketch of the problem and coordinate system.

Consider the flow of an incompressible Johnson-Segalman fluid in a curved channel of radius 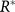 and uniform thickness 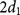 coiled in a circle with centre 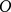 (see Fig. 1). We denote axial and radial directions by 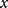 and 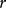 respectively. 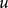 and 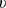 are the components of velocity in the axial and radial directions respectively. The shape of the propagated waves is given by
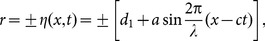
(1)where 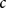 is the wave speed and 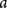 and 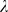 are the wave amplitude and wavelength respectively.

The equations which can govern the flow are:
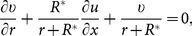
(2)

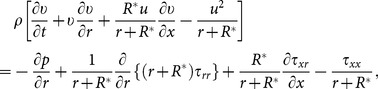
(3)

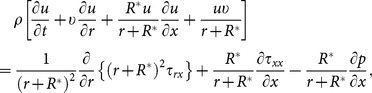
(4)


The Cauchy stress tensor 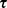 in a Johnson-Segalman fluid is [40]:
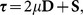


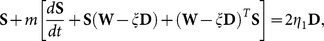
where the symmetric (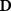) and skew symmetric (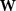) parts of velocity gradient are
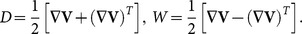



The above relations yield
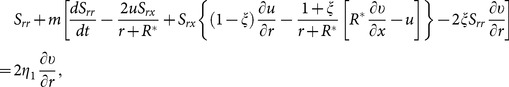
(5)

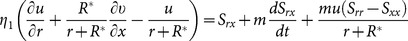


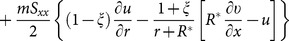


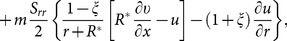
(6)

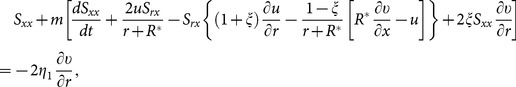
(7)where 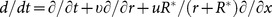 is the material time derivative, 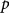 the pressure, 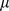 and 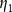 are the viscosities, 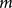 the relaxation time, 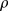 the density, 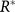 the curvature parameter, 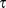 the elastic tension, 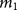 the mass per unit area, 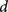 the coefficient of viscous damping, 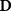 and 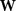 are the symmetric and skew symmetric parts of velocity gradient, 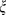 is the slip parameter and 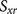, 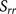 and 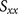 are the components of an extra stress tensor 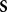.

The boundary conditions can be written as
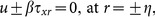
(8)

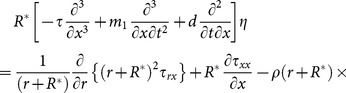


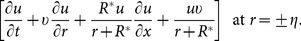
(9)


Defining
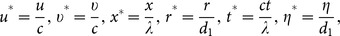


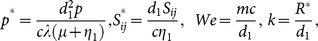

equations (3)–(9) become
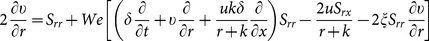


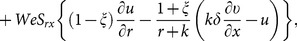
(10)

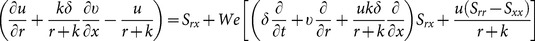


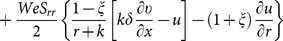


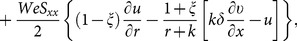
(11)

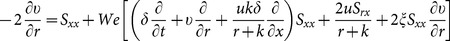


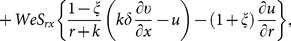
(12)

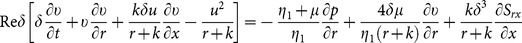


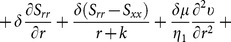


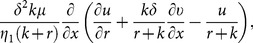
(13)

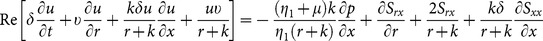


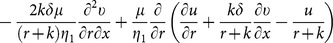


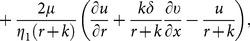
(14)with the boundary conditions
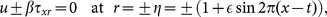
(15)

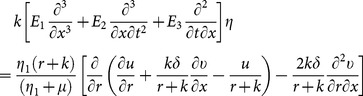


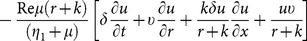


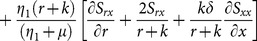


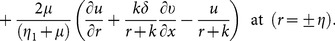
(16)


Note that the continuity equation (2) is satisfied identically, 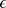 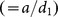 is the amplitude ratio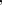 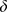 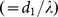 the wave number, 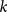 the dimensionless curvature parameter, 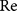 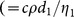) the Reynolds number, 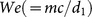 the Weissenberg number, 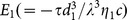 the wall tension parameter, 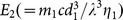 the mass per unit area parameter and 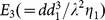 is the viscous damping parameter [30].

If 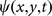 is the stream function then writing
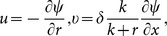
expressions (10)
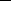
(16) after using long wavelength and low Reynolds number give
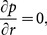
(17)

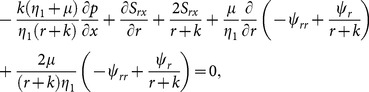
(18)

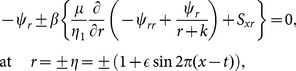
(19)

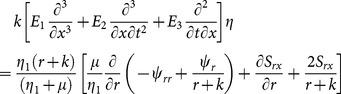


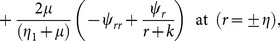
(20)with
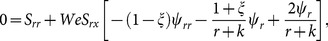
(21)

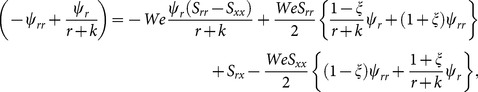
(22)

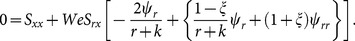
(23)


From Eqs. (21)–(23) one has [40]

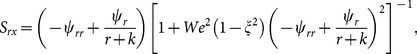
(24)

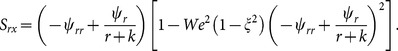
(25)


Due to Eqs. (17)–(18) one obtains
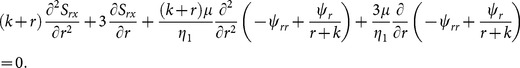
(26)


## Solution Procedure

Different from the work reported in [45], in which the authors have obtained the exact solutions, here the differential system is strongly non-linear and cannot be solved exactly. We therefore first proceed for the perturbation solution and write stream function and stress components as:
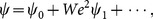
(27)

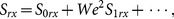
(28)

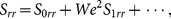
(29)

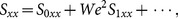
(30)


**(i) Zeroth order system**

Putting Eqs. (27) and (28) into Eqs. (19), (20), (25) and (26) and then equating the coefficients of 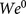 we arrive at
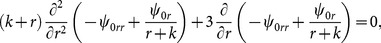
(31)

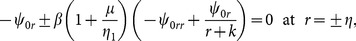
(32)

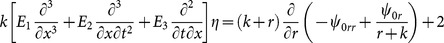


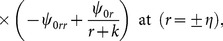
(33)


The solution of above equations are
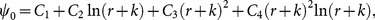
(34)

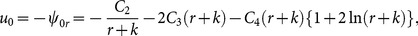
(35)where
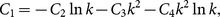


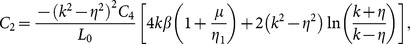


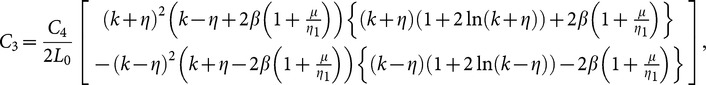


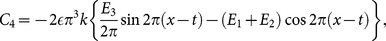


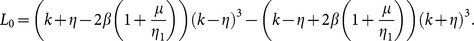



**(ii) First order system**

The coefficients of 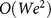 leads to the following expressions
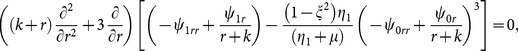
(36)

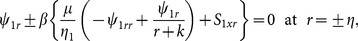
(37)

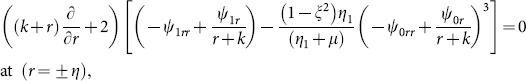
(38)

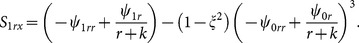
(39)


Putting the zeroth order solution expressions into first order system and then solving the resulting problems we have
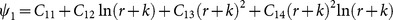


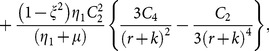
(40)

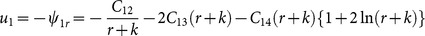


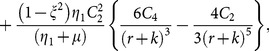
(41)

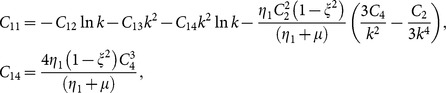


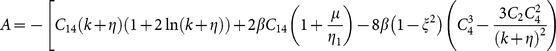


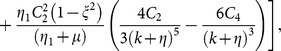


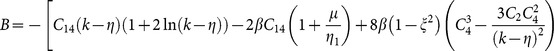


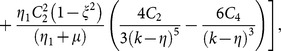


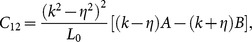


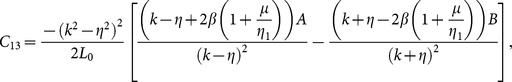
where 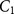 and 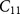 are obtained by the condition 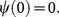


## Results and Discussion

In addition to the perturbation solutions, the governing Eq. (26) subject to the boundary conditions (19)and (20) have been solved for the stream function 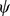 through the built in routine for solving nonlinear boundary value problems via Mathematica 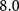. A comparative study between numerical and analytic solutions shows an excellent agreement. Moreover the obtained results are in a very good agreement with the existing studies in the limiting case 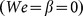.

**Figure 2:**
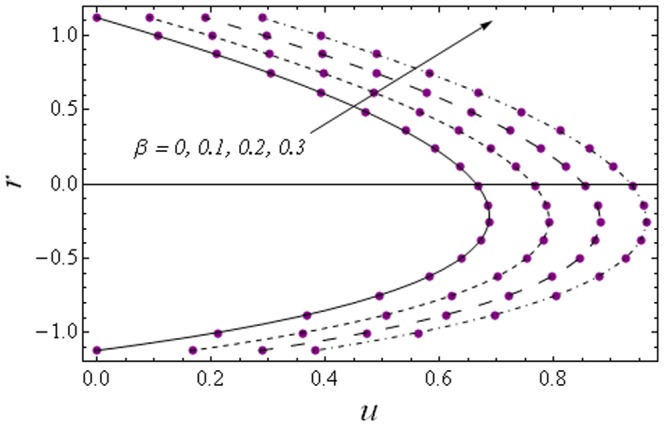
Comparison of numerical and analytic solutions 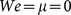 (Eq. (24)). Lines: Numerical solutions, Points: Analytic solutions.

**Figure 3:**
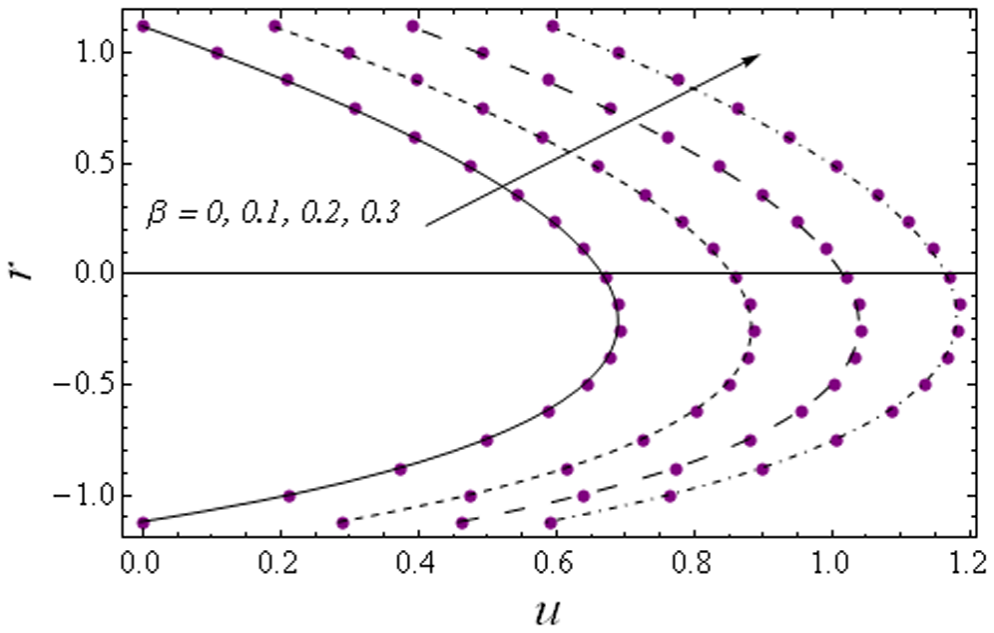
Comparison of numerical and analytic solutions 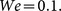 (Eq. (24)). Lines: Numerical solutions, Points: Analytic solutions.

**Figure 4:**
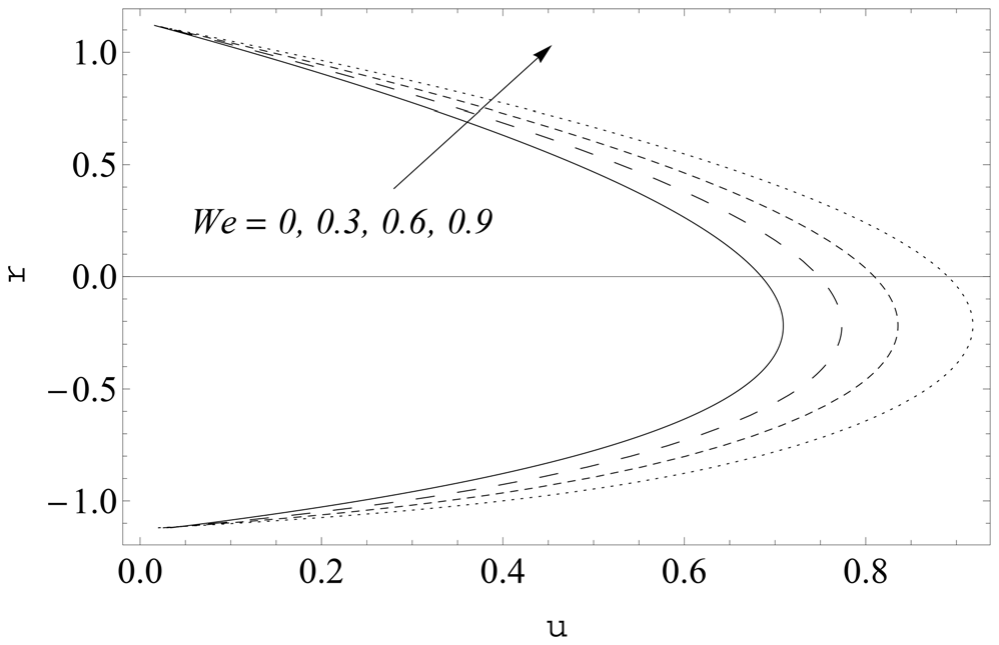
Influence of 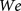 on 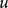 when 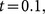 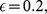 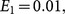 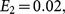 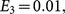 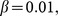 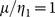 and 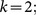 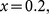 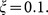

**Figure 5:**
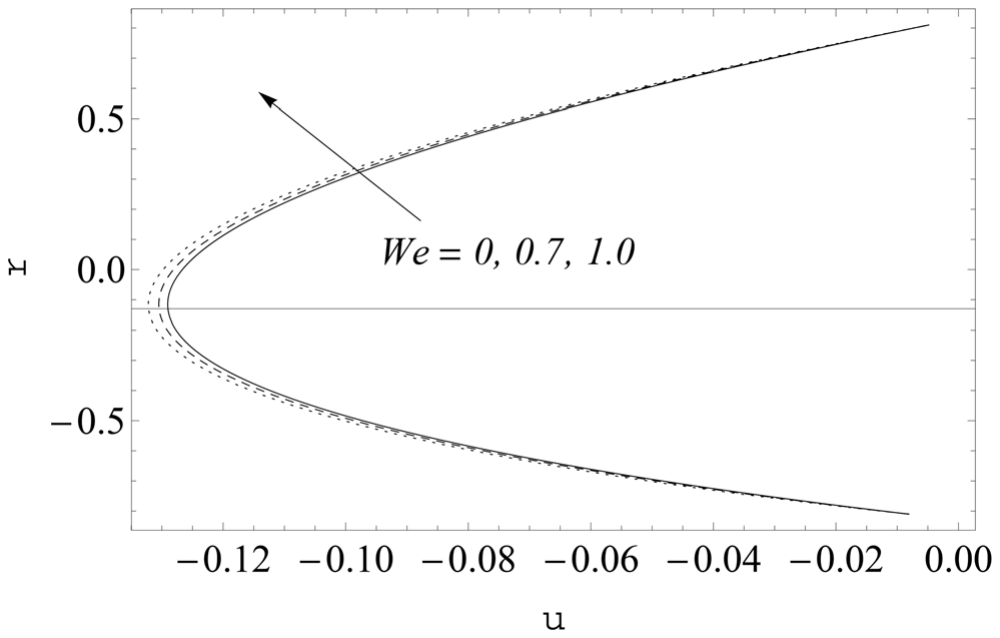
Influence of 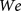 on 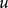 when 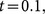 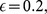 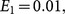 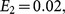 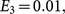 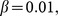 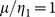 and 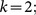 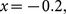 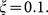

**Figure 6:**
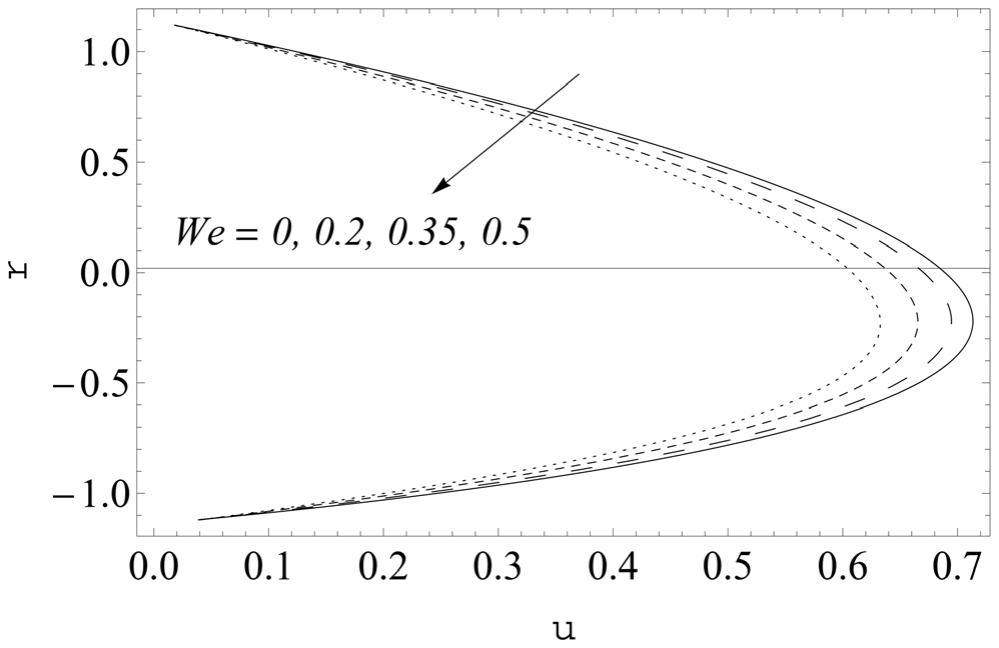
Influence of 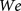 on 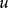 when 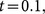 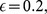 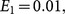 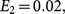 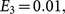 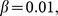 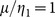 and 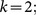 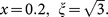

**Figure 7:**
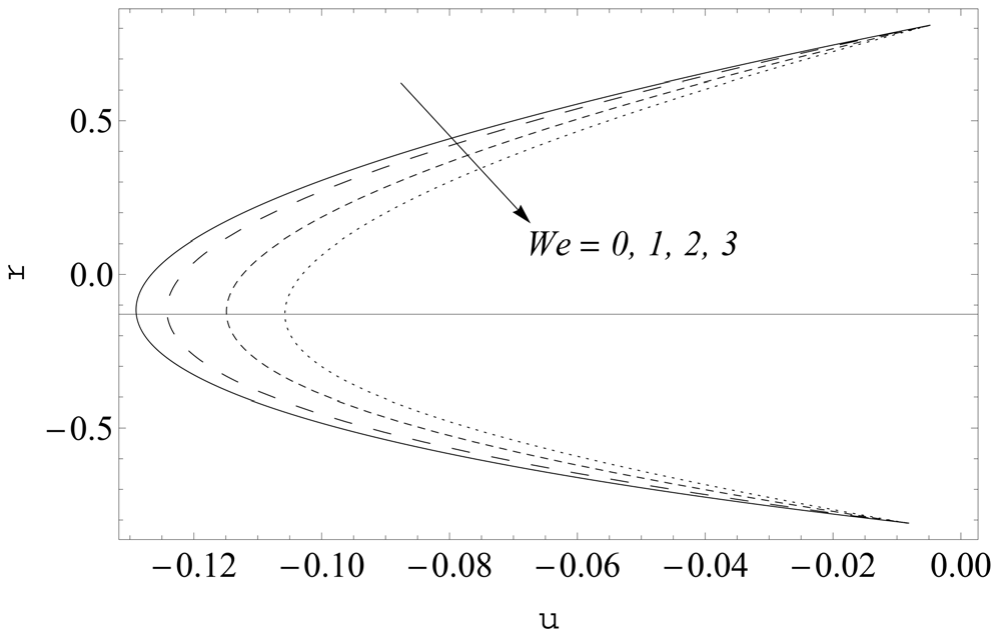
Influence of 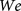 on 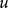 when 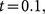 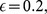 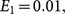 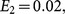 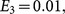 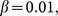 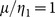 and 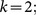 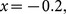 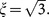

**Figure 8:**
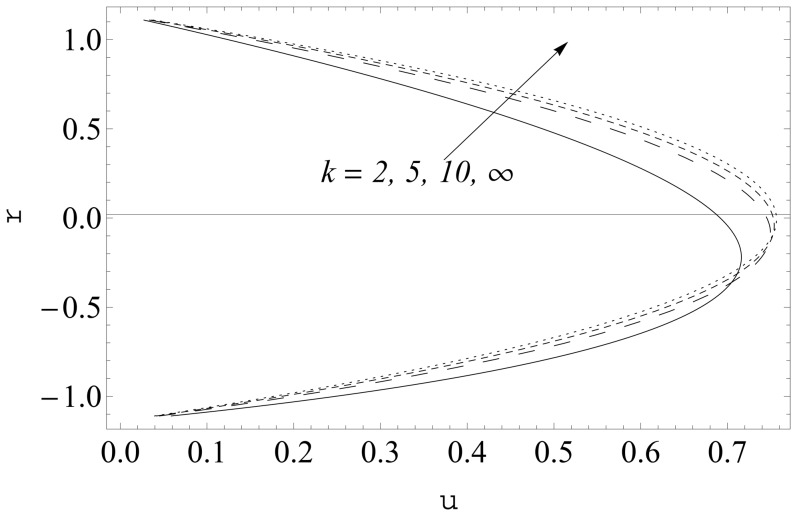
Influence of 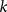 on 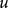 when 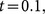 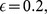 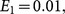 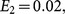 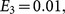 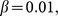 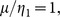 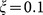 and 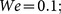 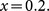

**Figure 9:**
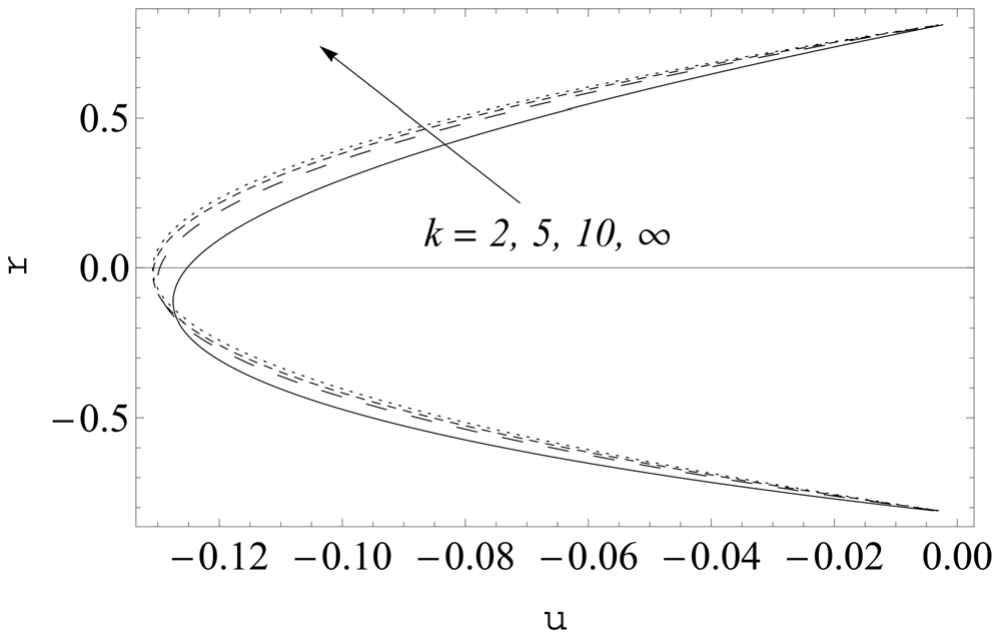
Influence of 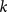 on 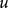 when 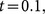 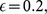 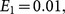 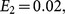 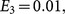 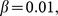 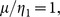 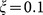 and 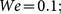 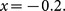

**Figure 10:**
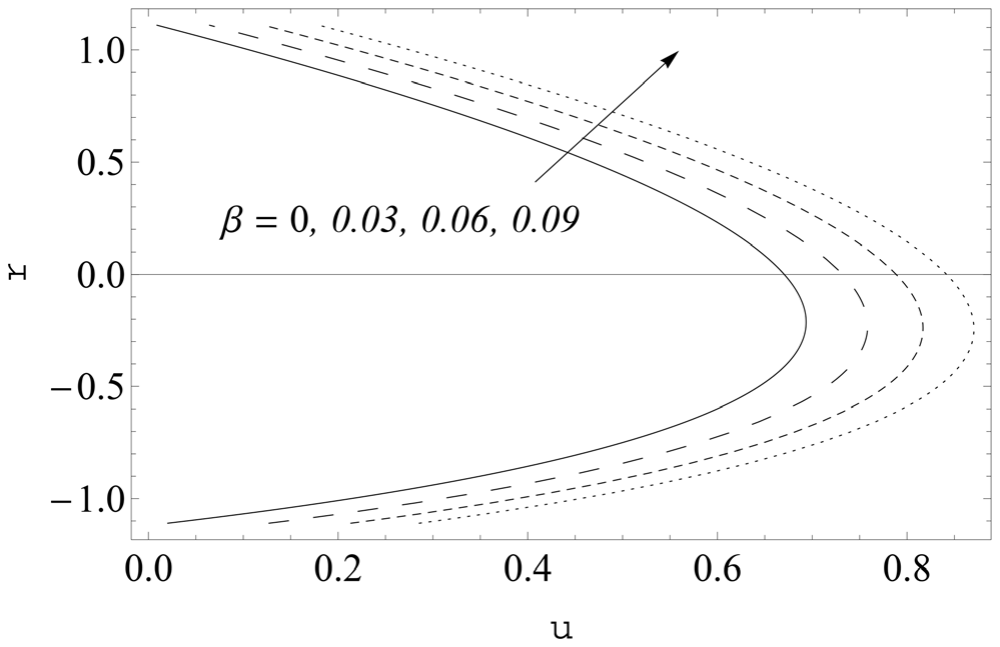
Influence of 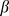 on 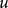 when 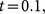 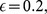 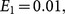 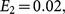 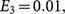 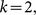 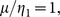 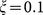 and 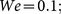 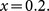

**Figure 11:**
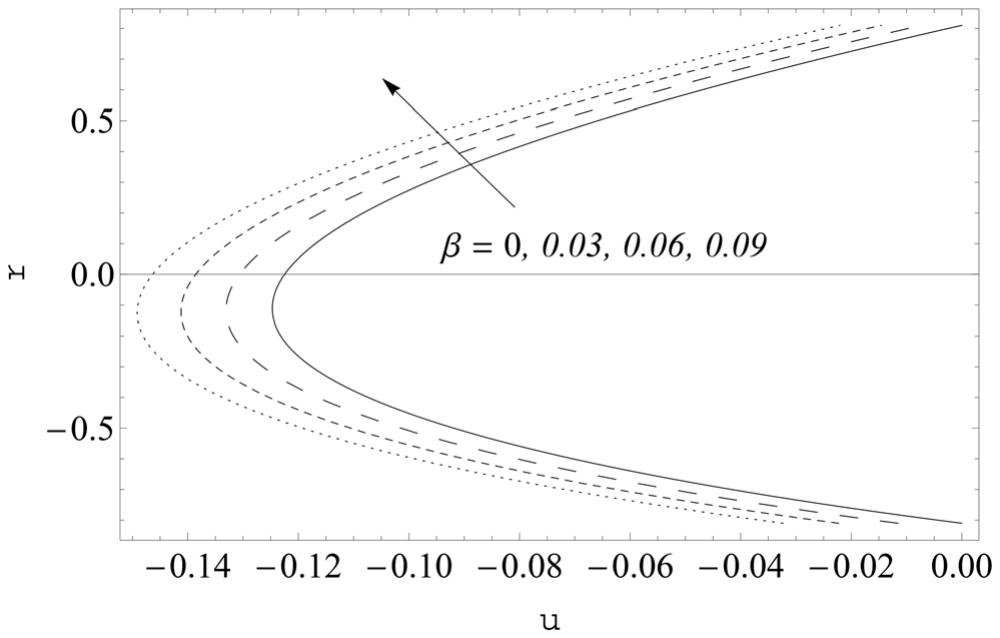
Influence of 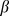 on 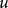 when 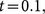 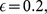 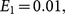 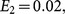 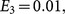 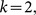 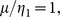 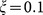 and 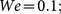 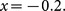

**Figure 12:**
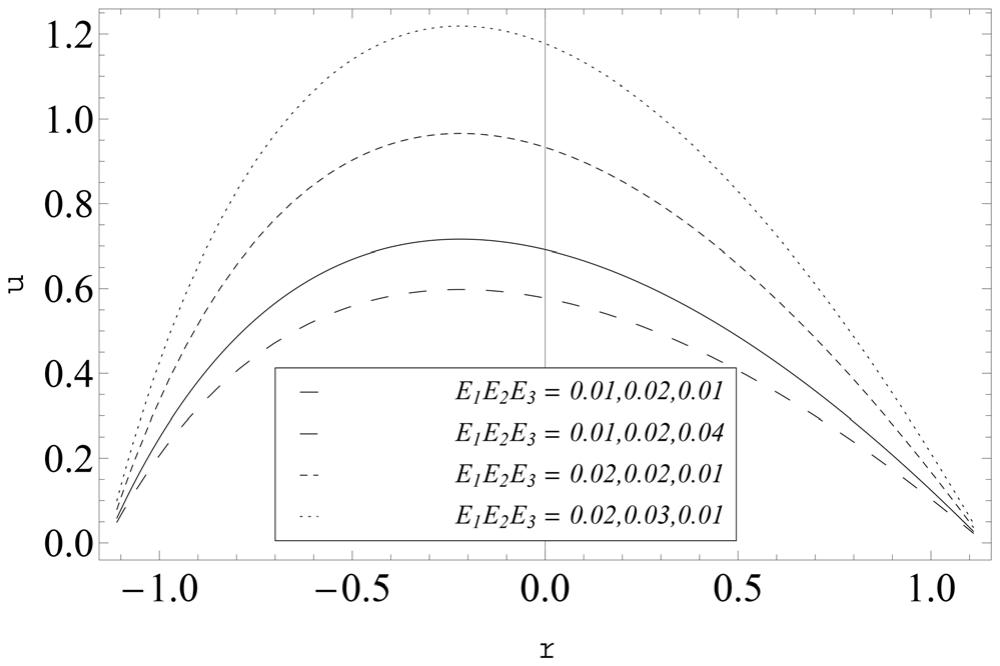
Influence of complaint wall parameters (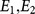 and 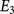) on 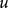 when 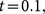 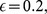 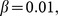 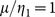 and 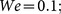 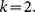

**Figure 13:**
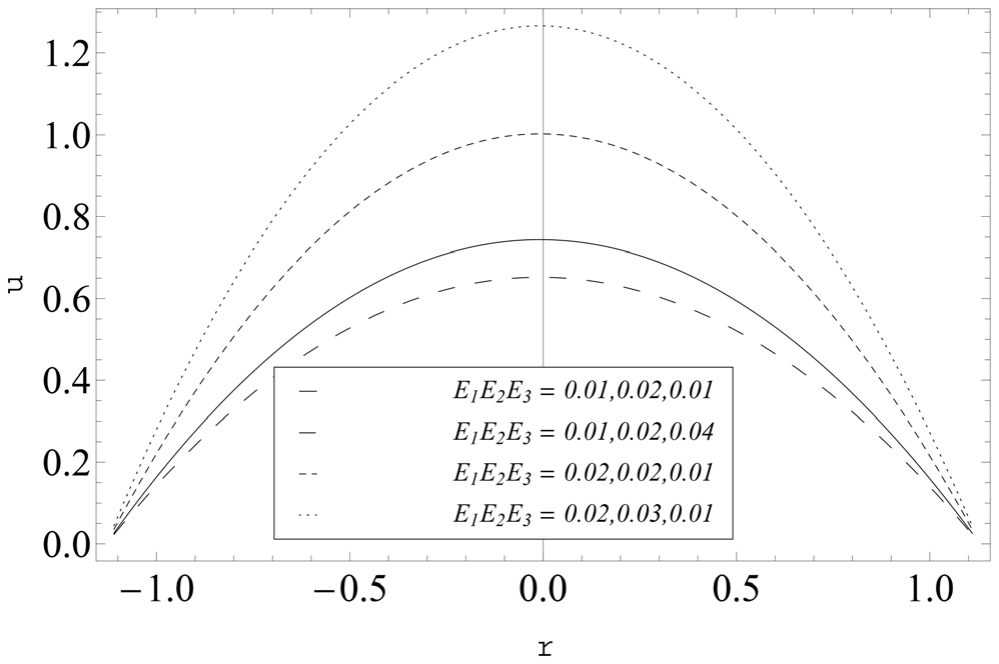
Influence of complaint wall parameters (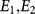 and 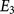) on 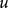 when 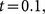 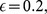 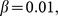 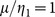 and 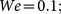 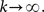

Here we focus on the behaviors of embedding parameters on the axial velocity 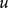 and stream function 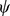. The numerical computations have been compared with the exact solutions for Newtonian fluid case (which can be obtained by setting 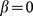 in the zeroth-order solution given in Eq. (35)) and with the perturbation solutions for small Weissenberg number 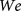 (see Figs. 2 and 3). It is noticed that the two sets of data are virtually similar demonstrating the validation of present results. Figs. 4–7 show the profiles of axial velocity for different values of Weissenberg number at two different cross-sections. It is clear that magnitude of axial velocity increases with an increase in the Weissenberg number 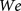 when 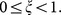 Further the maxima in the profiles in the Johnson-Segalman fluid is greater when compared with the Newtonian fluid (see Figs. 4 and 5). Figs. 6 and 7 plot the axial velocity profiles for different values of 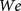 when 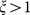. Here it is noticed that axial velocity is a decreasing function of 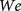. Moreover the deviation in the profiles is more significant in this case when compared with those obtained for 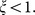 The effects of curvature parameter 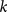 on the axial velocity are sketched in Figs. 8 and 9. Here the axial velocity profiles are shown at two different cross-sections It is noticeable that symmetry of the profiles about the central line is destroyed in the curved channel. However the profiles become symmetric about the central line for sufficiently large values of curvature parameter 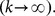 Moreover the axial velocity decreases near the lower wall and increases in the remaining part of the channel when there is an increase in 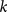. Further the maxima in the profiles in the curved channel lies below the maxima in the profiles for planar channel. Figs. 10 and 11 show the influence of slip parameter 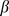 on the axial velocity 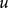. It is noticed that axial velocity is an increasing function of 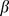. From the physical point of view an intensification in the slip effect leads to a larger magnitude of fluid velocity. Figs. 8–11 also indicate that profiles obtained at 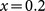 are largely affected than those obtained at 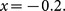 The compliant wall effects (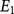,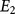 and 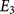) on the velocity are captured in the Figs. 12 and 13. It is seen that velocity is a decreasing function of 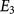. Further the axial velocity increases when there is an increase in 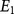 and 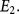 It is observed that the maxima in the profiles is tilted towards the central line of the channel for large curvature parameter 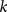 (see Fig. 13).

**Figure 14:**
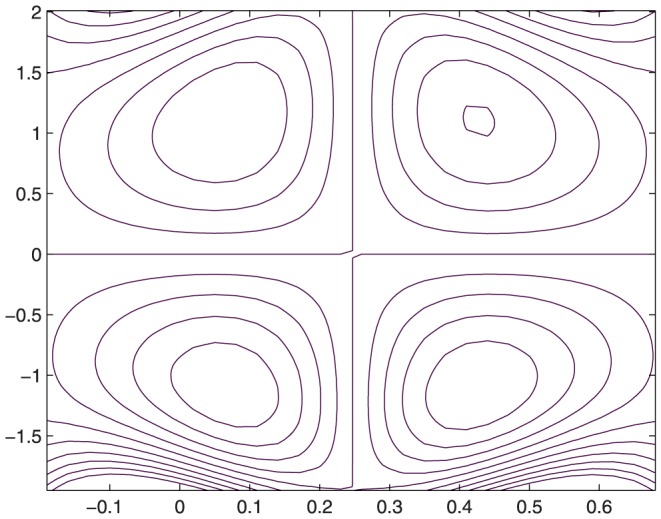
Influence of 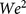 on 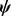 when 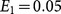, 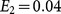, 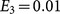, 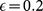, 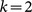; 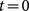, 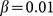, 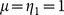, 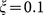; 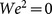.

**Figure 15:**
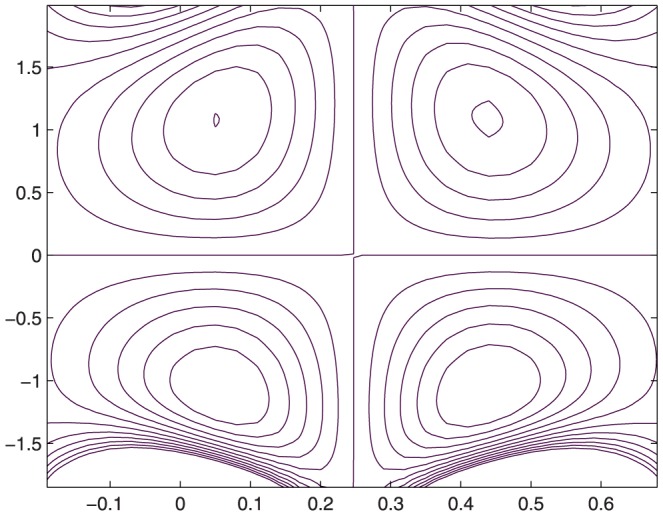
Influence of 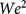 on 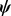 when 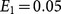, 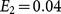, 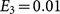, 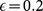, 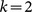; 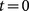, 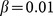, 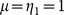, 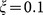; 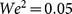.

**Figure 16:**
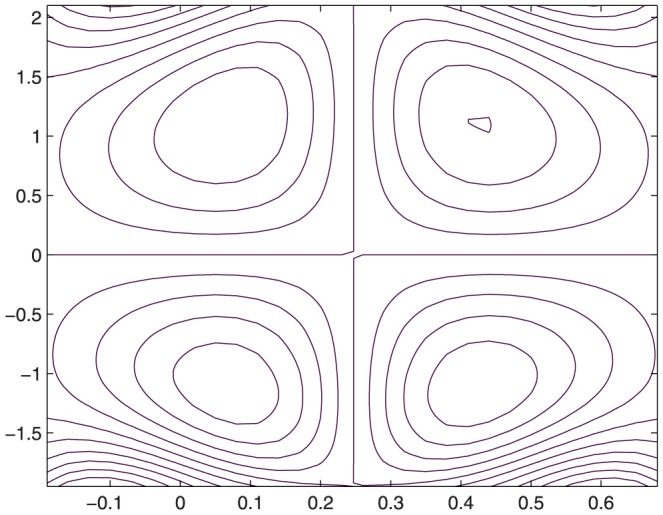
Influence of 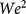 on 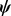 when 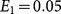, 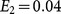, 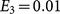, 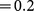, 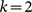; 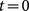, 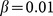, 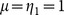, 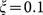; 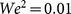.

**Figure 17:**
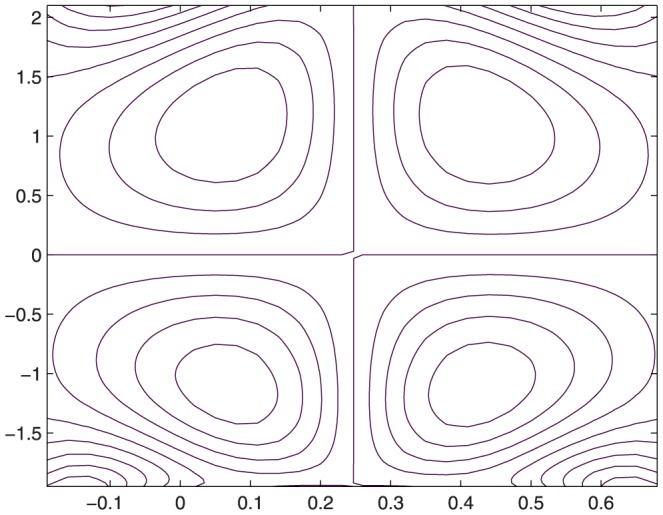
Influence of 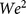 on 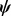 when 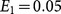, 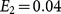, 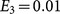, 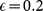, 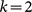; 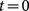, 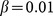, 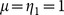, 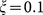; 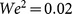.

**Figure 18:**
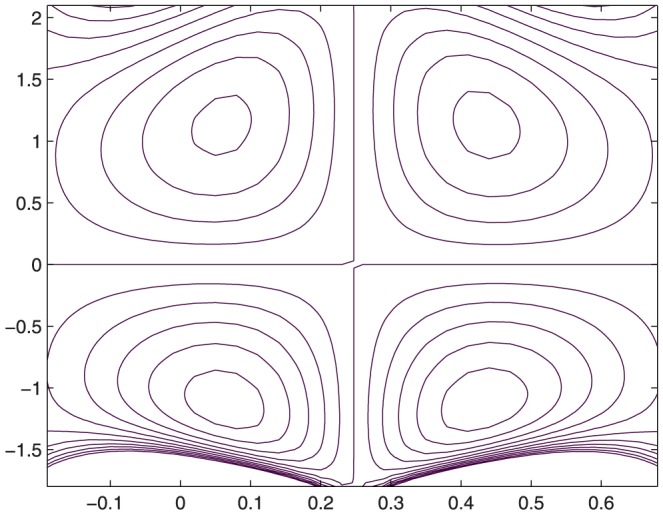
Influence of 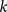 on 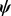 when 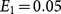, 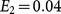, 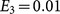, 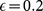, 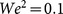, 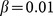, 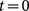 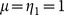, 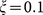; 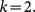

**Figure 19:**
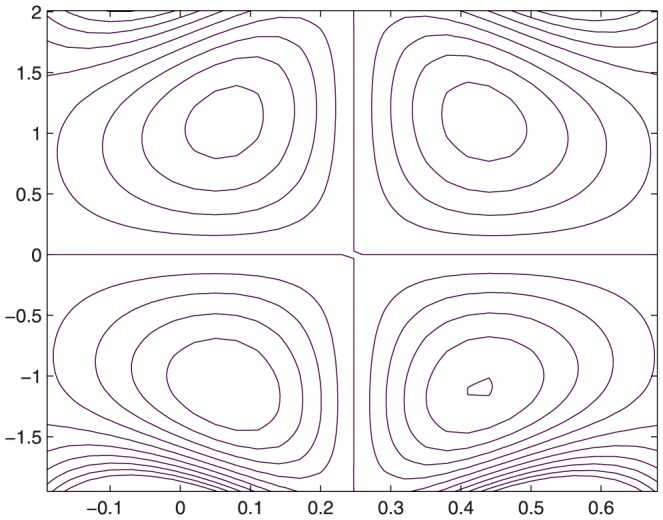
Influence of 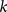 on 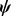 when 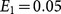, 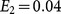, 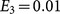, 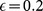, 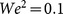, 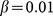, 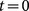 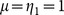, 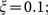 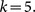

**Figure 20:**
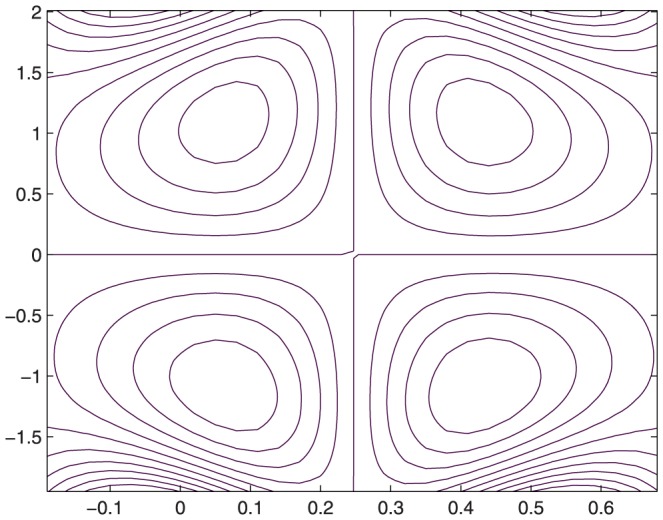
Influence of 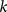 on 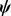 when 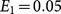, 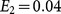, 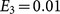, 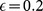, 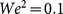, 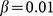, 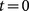 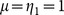, 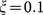; 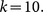

**Figure 21:**
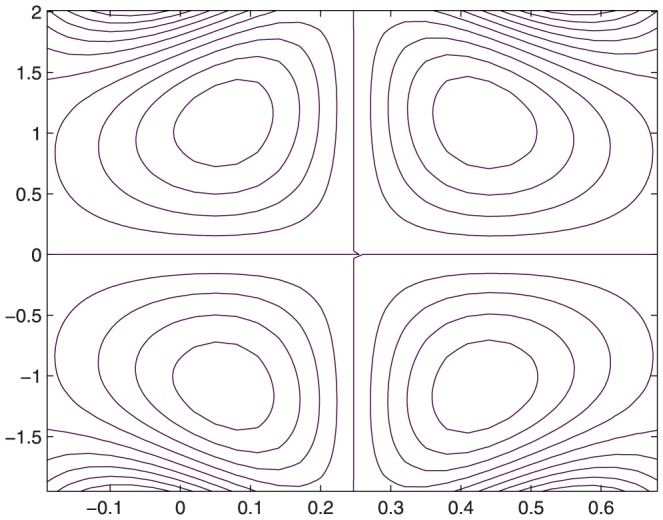
Influence of 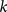 on 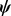 when 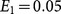, 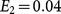, 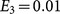, 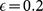, 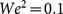, 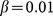, 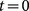 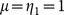, 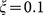; 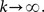

**Figure 22:**
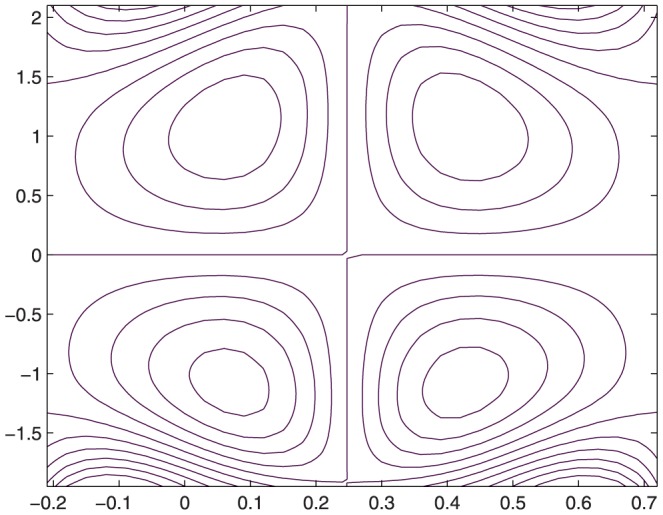
Influence of 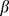 on 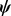 when 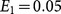, 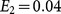, 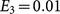, 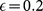, 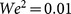, 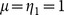, 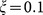, 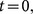 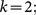 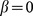.

**Figure 23:**
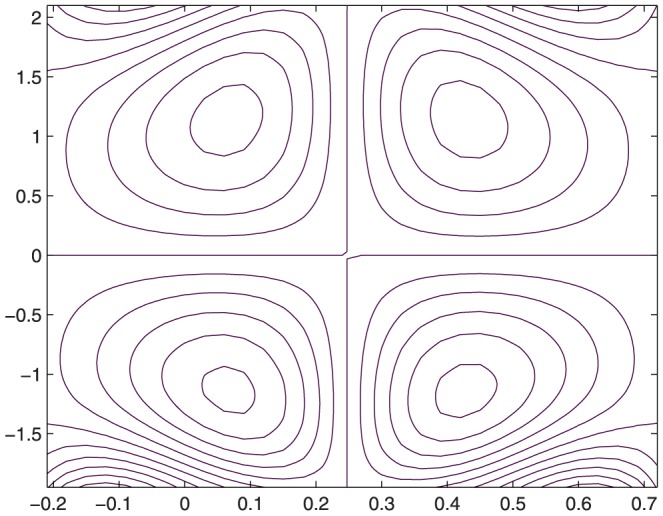
Influence of 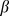 on 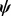 when 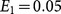, 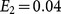, 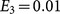, 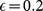, 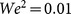, 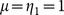, 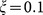, 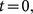 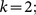 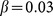.

**Figure 24:**
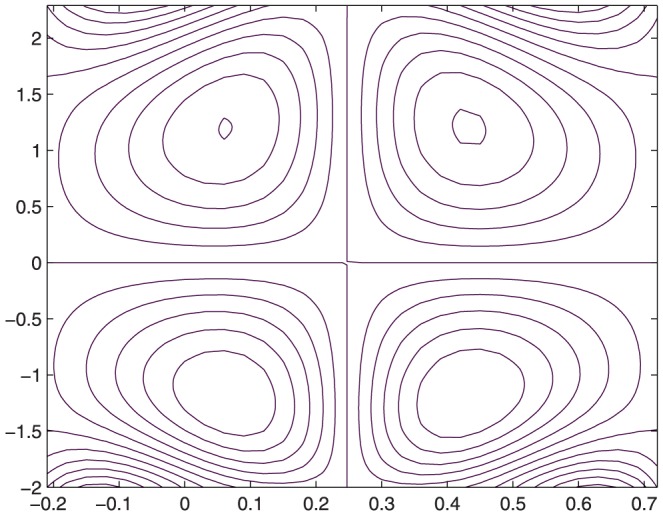
Influence of 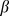 on 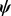 when 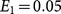, 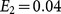, 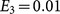, 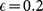, 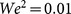, 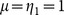, 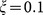, 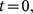 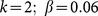.

**Figure 25:**
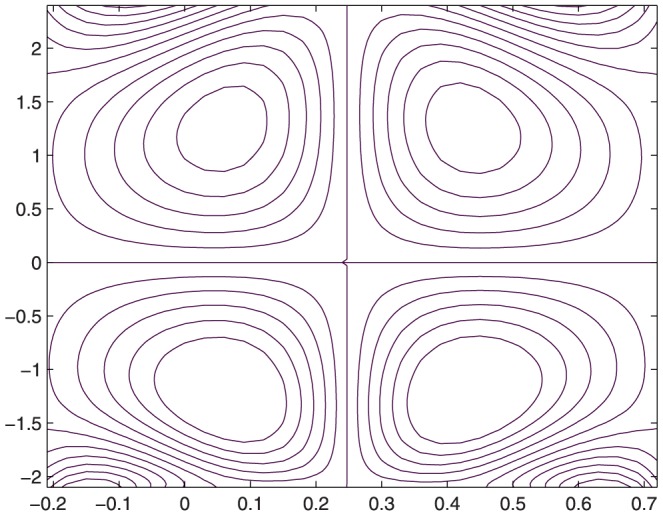
Influence of 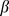 on 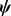 when 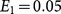, 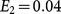, 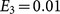, 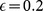, 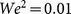, 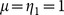, 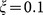, 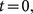 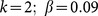.

The influence of all the parameters on the stream function 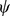 is observed in Figs. 14–25. Figs. 14–17 show that size and circulation of the trapped boluses increase with an increase in 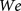 for 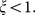 However for larger values of 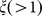 the size and circulation of the boluses decrease with an increase in 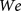. Moreover the boluses in the upper and lower parts of the channel are not symmetric. Stream lines for various values of slip parameter have been plotted in the Figs. 18–21. We notice that gradual increase in 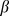 corresponds to an increase the size and circulation of the boluses. This is in fact due to the increase in the axial velocity with an intensification in the slip effect(noticed earlier in Figs. 10 and 11). Figs. 22–25 indicate that bolus size is different in the two halves of the channel for small values of curvature parameter 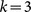, i.e. for curved channel; whereas the bolus attains its symmetric shape in the straight channel 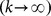. Interestingly the size and circulation of trapped bolus increases with an increase in 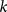 for 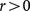. However for 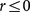 the circulation of the bolus is found to decrease upon increasing the curvature parameter.

## Conclusions

Influence of wall properties on the peristaltic transport of Johnson-Segalman fluid through a curved channel is investigated. Both analytic and numerical solutions of the developed differential system have been computed which are found in a very good agreement. It is found that velocity is an increasing function of 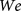 for 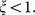 However it decreases with an increase in 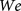 for 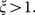 This reduction accompanies with the smaller size and circulation of boluses. It is noticed that magnitude of velocity in slip flow is greater than the no-slip flow. A significant increase in the velocity with an increase in the elastic parameters 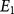 and 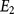 is noticed.
